# Diabetic Cheiroarthropathy in Type 1 Diabetes Mellitus and Coeliac Disease

**DOI:** 10.7759/cureus.31708

**Published:** 2022-11-20

**Authors:** Hiya Boro, Vikash Bundela, Vinita Jain, Velmurugan Mannar, Mazhar Dalvi

**Affiliations:** 1 Endocrinology, Aadhar Health Institute, Hisar, IND; 2 Gastroenterology, Aadhar Health Institute, Hisar, IND; 3 Pediatrics, Aadhar Health Institute, Hisar, IND; 4 Endocrinology, Aarupadai Veedu Medical College, Puducherry, IND; 5 Endocrinology, Mediclinic Al Noor Hospital, Abu Dhabi, ARE

**Keywords:** diabetic retinopathy, type 1 diabetes mellitus (t1d), diabetic hand syndrome, dupuytren's contracture, diabetic cheiroarthropathy

## Abstract

Diabetes mellitus can be associated with a variety of musculoskeletal disorders. Diabetic cheiroarthropathy or diabetic hand syndrome is one of the complications encountered in long-standing uncontrolled diabetes. It is characterized by limited movement of the joints of the hands along with thickening of the skin on the palmar and dorsal surfaces. There is an association between diabetic cheiroarthropathy and microvascular complications of diabetes, most commonly diabetic retinopathy. Early diagnosis of cheiroarthropathy can give the clinician an opportunity to screen for microvascular complications. Cheiroarthropathy is usually a clinical diagnosis. Treatment involves achievement of good glycemic control along with physiotherapy and occupational therapy.

We have described the case of a 16-year-old adolescent male with uncontrolled type 1 diabetes and coeliac disease who presented to us with diabetic cheiroarthropathy.

## Introduction

Diabetic cheiroarthropathy is a broad term that includes limited mobility of the joints of the hands [1]. There is thickening of the skin of the hands, referred to as diabetic sclerosis, pseudosclerodermatous hand of diabetes or diabetic stiff hand [1]. There is limited extension of the metacarpophalangeal (MCP) joints and the proximal (PIP) and distal interphalangeal (DIP) joints of the fingers, with fixed flexion contracture [2]. The skin over the palmar and dorsal surfaces of the hands becomes tight and waxy. Dupuytren's contracture, characterized by tightening of palmar fascia and formation of cords and nodules, can also be seen in long-standing diabetes.

Diabetic cheiroarthropathy can occur in long-standing diabetes. The prime pathophysiological mechanism includes formation of advanced glycation end products, cross linking of collagen and microangiopathy of blood vessels due to prolonged uncontrolled hyperglycemia [3]. Co-existence of coeliac disease can hamper diabetes management leading to poor glycemic control and higher incidence of complications. 

Here, we describe the case of an adolescent with long-standing uncontrolled type 1 diabetes mellitus (DM) and coeliac disease who presented with diabetic cheiroarthropathy.

## Case presentation

A 16-year-old adolescent male presented to our clinic with history of type 1 diabetes mellitus (DM) for the past 10 years, along with coeliac disease for the past eight years. He had poor glycemic control with a glycated haemoglobin level (HbA1c) of 16%. He was on pre-mixed twice daily insulin regimen. He was not adhering to strict gluten free and diabetic diet. He did not do self-monitoring of blood glucose. On physical examination, he was short with a height of 148 cm which was below the 5th percentile [4]. His weight was 34 kgs, which was also below the 5th centile [4]. He had evidence of microvascular complications in the form of distal symmetrical peripheral polyneuropathy, bilateral proliferative diabetic retinopathy and 2+ proteinuria, on urine examination that corresponded to 1.5 gm of proteins per day.

**Figure 1:**
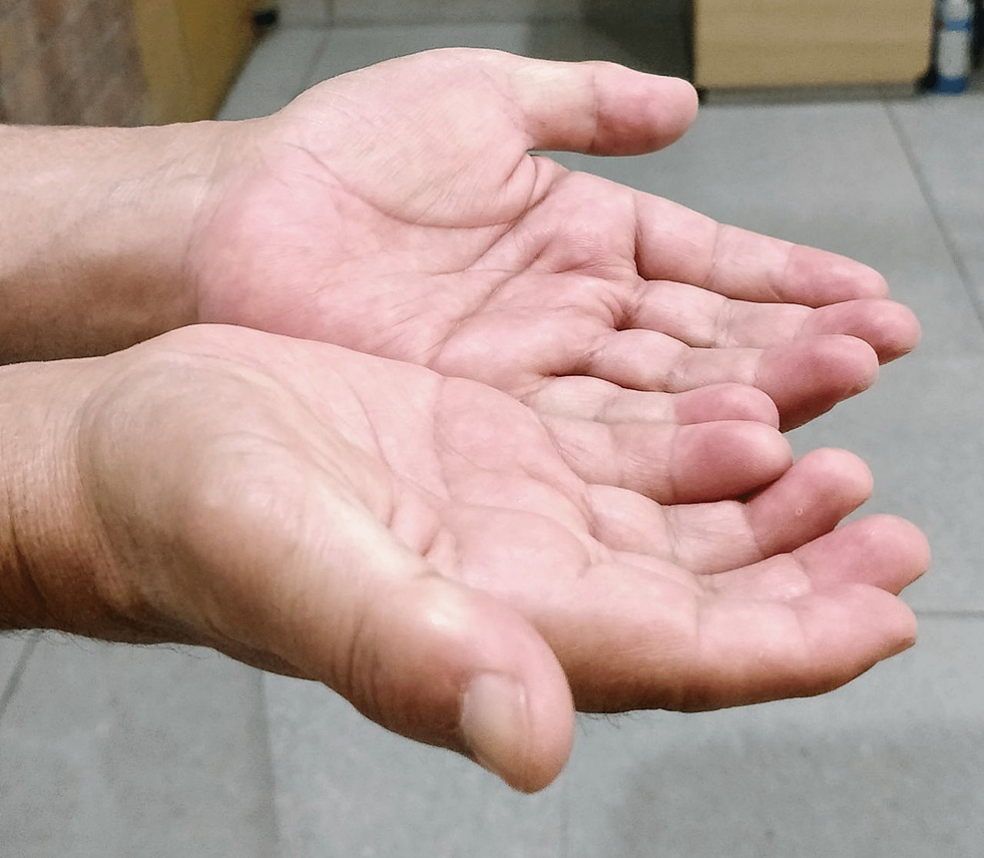
Fixed flexion contracture at the proximal and distal interphalangeal joints of the digits

**Figure 2:**
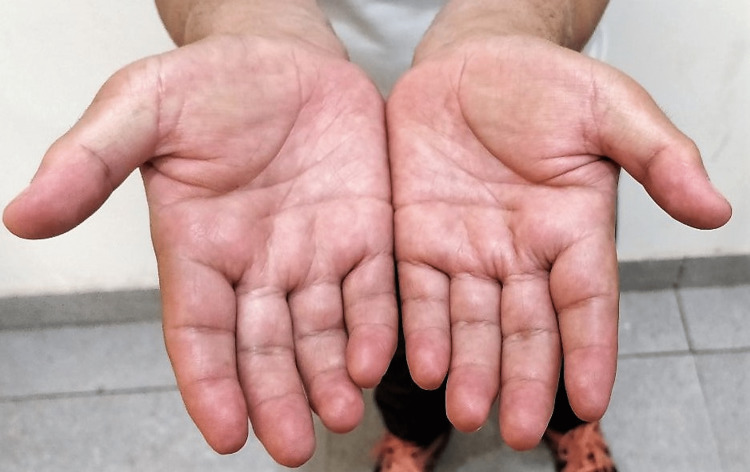
Thickening of the palmar surfaces of the hands

**Figure 3:**
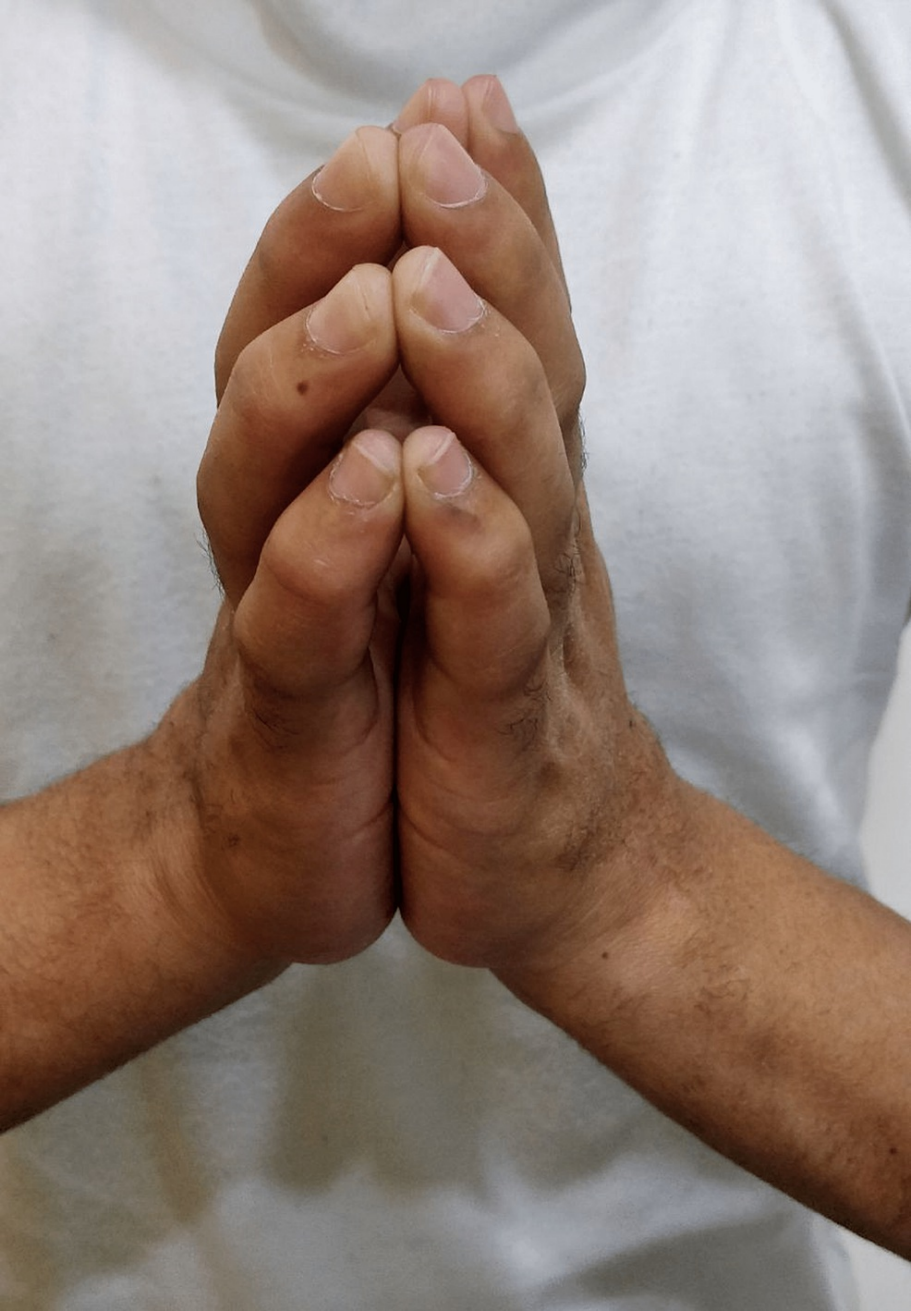
Positive Prayer’s sign

Patient complained of progressive painless stiffness of both his hands. He reported difficulty in carrying out fine activities like buttoning his shirt, writing, working on keyboard and injecting insulin. Hand examination showed the presence of diabetic cheiroarthropathy. This consisted of fixed flexion deformity at the level of PIP and DIP joints of both hands (Figure 1). There was thickening and induration of the skin of the palms (Figure 2). Prayer sign of the hands was positive, suggestive of limited joint mobility (Figure 3). Tinel’s and Phalen’s signs for carpal tunnel syndrome were negative. Neurological examination revealed impaired grip strength.

There was no history of trauma to the hands, no Raynaud’s phenomenon, no joint pain, no nodule at the joints. Radiographs of the hands did not reveal any significant finding apart from fixed flexion deformity at PIP and DIP joints. Rheumatological work up was negative with normal erythrocyte sedimentation rate (ESR), normal C reactive protein (CRP), negative rheumatoid factor (RA factor) and negative work up for scleroderma. Thyroid function tests of the patient revealed normal thyroxine level of 8.4 ug/dL (N:5.1-14.1 ug/dL), and normal thyroid-stimulating hormone (TSH) of 2.1 uIU/mL (N:0.27-4.2 uIU/mL). His anti-thyroid peroxidase (anti-TPO) antibodies were positive (anti-TPO 178 IU/L [N<34 IU/L]).

**Figure 4:**
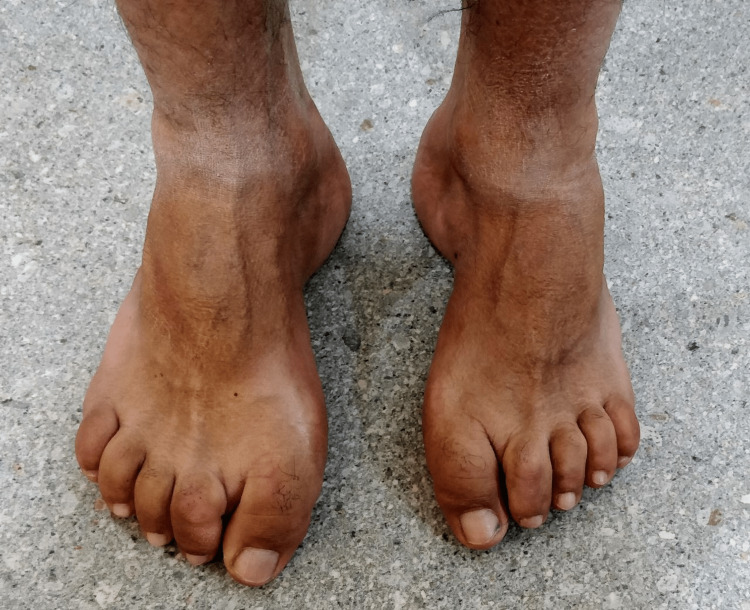
High risk feet with hallux valgus, hammer toes and exaggerated medial arches of feet

Foot examination showed presence of bilateral high risk feet with hammer toes, hallux valgus, pes cavus and itch marks (Figure 4). There was exaggeration of the medial plantar arches. There was no active ulceration of either foot.

Patient was provided counseling and education about diabetes and its complications. Patient was also educated about coeliac disease and counselled to maintain a gluten free diet. He was shifted to basal bolus insulin regimen for better glycemic control. He was referred to Ophthalmology department for panretinal photocoagulation for treatment of diabetic retinopathy. He was also initiated on angiotensin converting enzyme inhibitors (ACEI) for proteinuria.

He was educated about foot care for high-risk feet. He was also provided with customized footwear. For his hands, orthopaedic surgeon was consulted who advised conservative management in the form of strict glycemic control and stretching exercises.

Patient was referred for physical and occupational therapy. Patient benefited from daily stretching exercises of his hands and fingers. He reported a mild improvement in his joint movements after six months of continuous physiotherapy.

## Discussion

Cheiroarthropathy is often a neglected complication of diabetes [5]. It is a broad term that includes limited joint movements (LJM) of the hands and thickening of the palmar and dorsal fascia of the hands. Sometimes, the disease is progressive and may involve the shoulder or glenohumeral joint, which is then known as ‘shoulder hand syndrome.’

Diabetic cheiroarthropathy can be present in both type 1 and type 2 DM. Previous studies have shown that this complication is more common in type 1 DM [6-8]. One study has reported an overall prevalence of 30% [1], while a few others have quoted a prevalence of 8 to 50% [9,10]. The median duration of diabetes was more than 10 years in patients with type 1 DM. Typically, it is characterized by restriction of movements of the MCP, PIP and DIP joints. It usually starts on the ulnar side and spreads radially [2]. Presence of cheiroarthropathy can be easily manifested by simple bedside signs such as ‘Prayer sign’ and ‘Table Top sign’. For demonstration of Prayer sign, the patient is asked to hold both hands in opposition (as if praying), keeping the elbows fixed and the wrists extended. If the patient is not able to approximate the digits while praying, it is known as ‘Positive Prayer sign’. For demonstration of table top sign, patient is asked to touch the table with the palmar surfaces of the hands. If the patient is unable to keep his/her palms or digits completely flat on the table, it is known as ‘Positive Table Top sign’. Dupuytren’s contracture is characterized by thickening and tethering of the palmar surface of the hands, along with contracture of the digits, mainly involving the middle and ring fingers. The pathogenesis of diabetic cheiroarthropathy is multifactorial [11]. There is formation of advanced glycation end products (AGE) due to long-standing uncontrolled hyperglycemia [11]. There is formation of collagen cross links with extensive proliferation of collagen in skin, subcutaneous tissues, muscle and periarticular tissues. This collagen is also resistant to degradation. In addition, there is microangiopathy involving the blood vessels of the skin and subcutaneous tissues, leading to decreased blood supply [11]. The chronic low-grade ischaemia can lead to fibrosis of the skin, giving the appearance of tight, waxy skin.

Other common hand manifestations in long-standing diabetes can be carpal tunnel syndrome and tenosynovitis (trigger finger), ulnar neuropathy, and osteoarthritis of the first carpometacarpal joint [6].

Cheiroarthropathy can affect the feet as well characterized by impaired mobility of the toes and foot joints. There may be contracture and thickening of the plantar fascia and the skin over the toes. Tarsal tunnel syndrome can also be a common phenomenon in long-standing diabetes. The arches of the feet may be exaggerated and the patient may have difficulty walking along with risk of falling. Certain parts of the feet may be exposed to high pressure like the plantar surfaces of the great toes and the heels, increasing the risk of ulceration. 

Previous studies have shown that diabetic cheiroarthropathy has a correlation with the microvascular complications of diabetes. The most significant association is with diabetic retinopathy. This was first shown by Rosenbloom et al. in 1981 [8]. They reported a threefold increased risk of microvascular complications in patients with cheiroarthropathy. Subsequent studies have also proved this association [12,13]. Our patient had evidence of retinopathy, neuropathy and nephropathy in addition to diabetic cheiroarthropathy.

The diagnosis of diabetic cheiroarthropathy is usually clinical. However, a few imaging modalities like ultrasound and magnetic resonance imaging (MRI) may show thickening of flexor tendon sheaths, as reported in a few studies [14,15].

Treatment for diabetic cheiroarthropathy involves achieving good glycemic control. Lister et al. reported resolution of diabetic cheiroarthropathy with intensive glycemic control in type 1 diabetes [16]. Hider et al. reported resolution of the condition after pancreas transplantation in type 1 diabetes [17]. Physiotherapy and occupational therapy play an important role in the management. Surgical interventions like carpal tunnel release surgery and glucocorticoid injections into fingers are more helpful in cases of associated carpal tunnel syndrome. 

Novel therapies targeting advanced glycation end products have been tried in animal models, but are still not recommended for clinical use in humans due to safety concerns and lack of evidence. These include alagebrium (ALT 711), aminoguanidine, pyridoxamine, and benfotiamine.

## Conclusions

We have described a case of diabetic cheiroarthropathy in an adolescent male with a long-standing history of type 1 DM and coeliac disease. Diabetic cheiroarthropathy has significant association with microvascular complications. Achievement of good glycemic control is the mainstay of management, along with physiotherapy and occupational therapy. More research is required to develop targeted therapy for curative management of cheiroarthropathy.
